# Innate Immune Evasion Mediated by Flaviviridae Non-Structural Proteins

**DOI:** 10.3390/v9100291

**Published:** 2017-10-07

**Authors:** Shun Chen, Zhen Wu, Mingshu Wang, Anchun Cheng

**Affiliations:** 1 Institute of Preventive Veterinary Medicine, Sichuan Agricultural University, Chengdu 611130, China; zhenwu@stu.sicau.edu.cn (Z.W.); mshwang@163.com (M.W.); 2 Research Center of Avian Disease, College of Veterinary Medicine of Sichuan Agricultural University, Chengdu 611130, China; 3 Key Laboratory of Animal Disease and Human Health of Sichuan Province, Chengdu 611130, China

**Keywords:** *flavivirus*, HCV, non-structural protein, immune evasion, emergence

## Abstract

Flaviviridae-caused diseases are a critical, emerging public health problem worldwide. Flaviviridae infections usually cause severe, acute or chronic diseases, such as liver damage and liver cancer resulting from a hepatitis C virus (HCV) infection and high fever and shock caused by yellow fever. Many researchers worldwide are investigating the mechanisms by which Flaviviridae cause severe diseases. Flaviviridae can interfere with the host’s innate immunity to achieve their purpose of proliferation. For instance, dengue virus (DENV) NS2A, NS2B3, NS4A, NS4B and NS5; HCV NS2, NS3, NS3/4A, NS4B and NS5A; and West Nile virus (WNV) NS1 and NS4B proteins are involved in immune evasion. This review discusses the interplay between viral non-structural Flaviviridae proteins and relevant host proteins, which leads to the suppression of the host’s innate antiviral immunity.

## 1. Introduction

**Figure 1:**
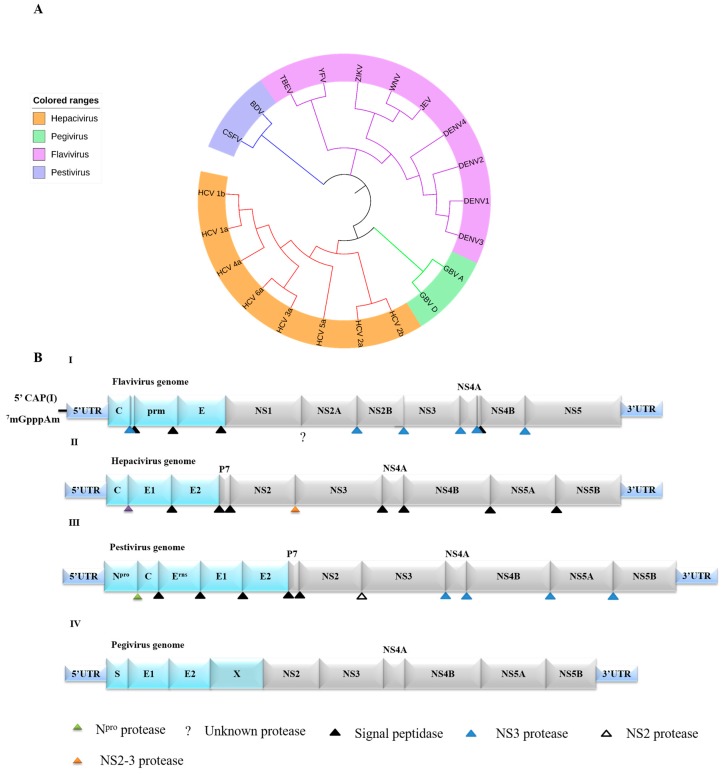
Flaviviridae phylogenetic characteristics and basic features of Flaviviridae reference genomes. (**A**) Flaviviridae family tree. Viruses related to this review and other representative Flaviviridae viruses; (**B**) The viral genome is shown in a schematic representation with a single long ORF (open reading frame) encoding the polyprotein, which is flanked by a 5′-terminal non-coding region (NCR) and a 3′-terminal NCR. The amino terminus of the genome encodes 3~5 structural proteins that form the virus particle and 7 non-structural (NS) proteins. Light blue and grey bars represent the structural and NS proteins, respectively. Cleavage sites for signal peptidase (black triangle), NS3 protease (blue triangle), unknown protease (?), NS2 protease (white triangle), N^pro^ protease (green triangle), and NS2-3 protease (yellow triangle) are indicated.

Flaviviridae viruses are responsible for many important diseases that affect public health worldwide. According to the latest International Committee on Taxonomy of Viruses (ICTV) report, Flaviviridae currently comprise the following four genera: *Flavivirus*, *Hepacivirus*, *Pegivirus* and *Pestivirus* (Figure 1A). The Flaviviridae viruses include yellow fever virus (YFV), Zika virus (ZIKV), Japanese encephalitis virus (JEV), West Nile virus (WNV), hepatitis C virus (HCV) and dengue virus (DENV). YFV, ZIKV, JEV, WNV and DENV are the leading causes of arthropod-borne human diseases worldwide [1]. HCV is a bloodborne virus that is commonly transmitted through unsafe injection practises, inadequate sterilization of medical equipment, and transfusion of unscreened blood and blood products [2]. Globally, between 130 and 150 million people suffer from chronic HCV infections. More than one-third of the world’s population live in areas at risk for DENV infection, and an estimated 400 million people are infected with DENV yearly [3]. WNV is common in Africa, Europe, the Middle East and North America, and WNV can develop into a serious neurological illness. Fortunately, approximately 80% of infected people are asymptomatic [4].

## 2. Genome and Life Cycle of Flaviviridae

The Flaviviridae family includes a group of enveloped RNA viruses that contain a single-stranded positive-sense RNA genome approximately 9.4~13 kb in length [5]. The Flaviviridae genome is composed of a polyprotein precursor flanked by a 5′-terminal non-coding region (NCR) and a 3′-terminal NCR. The polyprotein is processed by viral and host-cell proteases to produce approximately 10~12 mature proteins (including structural and non-structural proteins) (Figure 1). The amino terminus of the genome encodes 3~5 structural proteins that form the virus particle, and the remaining genome encodes 7 non-structural proteins essential for viral replication (Figure 1). Throughout the life cycle of the *flavivirus*, the E protein interacts with cellular receptors, allowing the virions to absorb onto the cell surface and enter the cells through receptor-mediated endocytosis [6]. The acidified environment of the endosomes enables the fusion of the viral and endosomal membranes [7,8]. Once the genome is released into the cytoplasm, the positive strand RNA is translated into a polyprotein that is processed by viral and host proteases. *NS*3 and *NS*5, which act as a serine protease and an RNA-dependent RNA polymerase, respectively, form a viral replication complex with other proteins [6,9]. Subsequently, the virus assembles on the surface of the endoplasmic reticulum (ER), and viral particles pass through the Golgi apparatus and are released by exocytosis [7].

## 3. Innate Sensing of Viruses by Pattern Recognition Receptors

**Figure 2:**
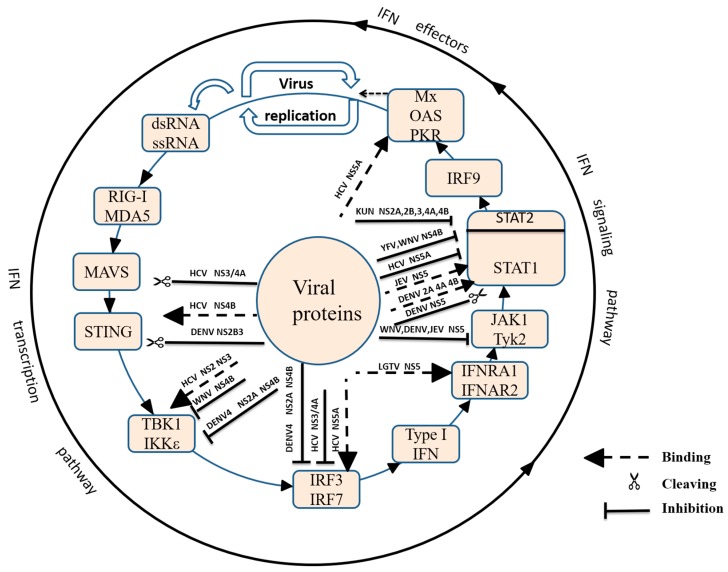
Suppression of type I IFN production by Flaviviridae viruses. Toll-Like receptors (TLRs) and retinoic acid-inducible gene I (RIG-I) receptors are activated by the virus; host antiviral responses are then activated, leading to the production of type I IFN. The binding of type I IFN to its cognate IFNARs activates the Janus kinase (JAK)-signal transducer and activator of transcription factor (STAT) signalling pathway. Secreted type I IFNs bind their cognate heterodimeric receptors (IFNAR1 and IFNAR2) and induce Tyk2 and Jak1 kinase activation, leading to the formation of the ISGF3 transcription factor complex. The complex translocates into the nucleus and induces the transcription of antiviral IFN-stimulated genes (ISGs). Flaviviridae have developed multiple strategies to escape or counteract host immune responses, including cleaving mitochondrial antiviral signaling (MAVS) by hepatitis C virus (HCV) NS3/4A; competing with MAVS for binding to stimulator of IFN genes (*STING*) to affect RIG-I-like receptors (RLRs) signalling transduction by HCV NS4B; cleaving STING by dengue virus (DENV) NS2B3; interacting with TANK-binding Kinase (TBK1) by HCV NS2 and NS3; inhibiting TBK1 phosphorylation by West Nile virus (WNV) NS4B and DENV4 NS2A and NS4B; inhibiting interferon regulatory factor-3 (IRF3) phosphorylation by DENV4 NS2A and NS4B and HCV NS3/4A; inhibiting interferon regulatory factor-7 (IRF7) activation by interacting with IRF7; interacting with IFNAR1 by Langat virus (LGTV) NS5; reducing Tyk2 phosphorylation by Japanese encephalitis virus (JEV), DENV and JEV NS5; interacting with STAT1 by DENV NS2A, NS4A and NS4B and JEV NS5; cleaving STAT1 by DENV NS5; inhibiting STAT1 phosphorylation by HCV NS5A, yellow fever virus (YFV) and WNV NS4B; inhibiting STAT2 phosphorylation by Kunjin virus (KUNV) NS2A, NS2B, NS3, NS4A and NS4B; and suppressing double-stranded RNA-activated protein kinase (PKR) and 2’-5’ OAS function by HCV NS5A.

Viruses are recognized by different types of pattern recognition receptors (PRRs) as pathogen-associated molecular patterns (PAMPs). PRRs, such as the Toll-Like receptors (TLRs) and retinoic acid-inducible gene I (RIG-I)-like receptors (RLRs), are activated by PAMPs, resulting in the activation of transcription factors and the production of cytokines, such as interleukin and interferon [10] (Figure 2). Of the PRRs, the most important receptors for Flaviviridae are *RIG-I/MDA5* (melanoma differentiation-associated gene 5) and TLR3. TLR3 can mediate the activation of immune cells, including dendritic cells, macrophages, and B cells [11], and can be activated by recognition of dsRNA, which further recruits and activates the adapter protein Toll/IL-1 receptor (TIR) domain-containing adaptor TRIF [12]. Stimulation of the TLR3-TRIF signalling pathway activates the transcriptional factors NF-κB and interferon regulator factor 3/7 (IRF3/7), which subsequently results in the translocation of NF-κB and IRF3/7 into the nucleus and the production of various cytokines, such as type I Interferon (IFN) [13]. RIG-I and MDA5 are activated by the recognition of short double-stranded RNA (dsRNA) or 5′ triphosphorylated dsRNA in the cytoplasm, and their activation triggers homo-oligomers that bind mitochondrial antiviral signaling (MAVS, also known as Cardif, VISA or IPS-1)/TRAF3 [14,15,16,17] and that then activate TANK-binding Kinase (TBK1) [18,19,20,21]. Phosphorylated TBK1 activates IRF3 or IRF7, and the activated transcriptional factors translocate into the nucleus and bind ISREs (IFN-stimulated response elements) to induce type I IFN mRNA transcription (Figure 2) [21,22,23,24,25]. Secreted type I IFNs bind their cognate heterodimeric receptors (type I interferon receptor (IFNAR1) and type II interferon receptor (IFNAR2)) and induce the downstream Janus kinase (JAK)-signal transducer and activator of transcription factor (STAT) signalling pathway and the transcription of antiviral IFN-stimulated genes (ISGs) (Figure 2) [26]. Recently, a stimulator of IFN genes (*STING*), also known as *TMEM173/MPYS/ERIS/MITA*, was identified as an adaptor that functions downstream of MAVS and upstream of TBK1 [27,28]. STING is a key component in the activation of IRF3 and NF-κB, and Kumar and colleagues have shown that STING-deficient mice cannot produce sufficient type I IFN to inhibit RNA viral infection [29].

The activation of the PRRs mediates the host antiviral immunity and results in the release of several cytokines that inhibit virus replication. IFN can effectively inhibit the virus, but the specific molecular mechanisms require further clarification. Interestingly, DENV replication was inhibited in cells pre-treated with IFN; however, the IFN treatment of cells after DENV infection did not produce an effective antiviral effect [30]. These studies suggest that the virus has developed mechanisms to antagonize the IFN-mediated immune response. By 1999 [31], the immune escape of Flaviviridae was confirmed, but the different viral proteins of different Flaviviridae have dissimilar immune escape mechanisms. Understanding the mechanism by which Flaviviridae escape the immune system and result in increased disease severity is important for the design of novel therapies at the immunological level. The non-structural proteins of Flaviviridae play an important role in immune escape. Here, we summarize the current research progress regarding immune evasion mediated by Flaviviridae non-structural proteins.

## 4. Flaviviridae Antagonism, IFN Induction and IFN-Dependent Signalling Pathways

### 4.1. Non-Structural Protein 1 (NS1) Inhibits the TLR Signalling Pathway

*NS1* of Flaviviridae is an essential gene that generates a highly conserved ca. 48 kDa glycoprotein that is localized to the lumen of the ER by a signal sequence located at the C-terminus of the structural envelope protein E [32]. NS1 is thought to be involved in the viral RNA replication process and the development of disease, because a deletion of the first YFV NS1 glycosylation site reduces the amount of viral replication and inhibits the secretion of NS1 [33]. The glycosylation site (Asn-207) and Cys residues of DENV NS1 is important in viral life cycle. Mutants at Cys sites (Cys4, Cys55, Cys291) and glycosylation site (Asn-207) significantly impaired Viral replication [34]. The mature E protein binds to the NS1 protein, and is not caused by incomplete hydrolysis of the precursor protein, suggesting that the complex is a nonfunctional non-specific protein in the viral replication cycle [35]. The HCV and pestivirus P7 proteins are counterparts of Flaviviridae NS1 (Figure 1) with a lower molecular weight. E2, P7 and NS2 can form a fusion protein, suggesting that P7 may play an important role in glycoprotein maturation [36,37]. During in vivo Flaviviridae infection, the expression levels of NS1 are high. Inoculation with the NS1 protein can induce a protective immune response against Flaviviridae infection, and thus, the NS1 protein is one of the primary immunogens during viral infection [38,39].

As we all known, DENV can cause fatal vascular leak. The cause of this disease may be associated with an excessive immune response. DENV-NS1 and the innate immune response are inextricably linked, on the one hand through the TLR4 receptor, activation of innate immune response [40,41], on the other hand, the NS1 protein is thought to inhibit the innate antiviral immunity primarily by interfering with the TLR signalling pathway, Wilson and colleagues were the first to report that WNV can inhibit TLR3-dependent IRF3 transcription in WNV replicon-bearing cells. Because the stimulation of TLR3 can induce NF-κB activation, the authors investigated whether WNV can negatively regulate the NF-κB activation [42]. Using reporter assays, the authors determined that the NF-κB-Luc activity was inhibited in either HeLa WNV replicon-bearing cells or 293/TLR3 cells [42]. Because Flaviviridae NS proteins can inhibit the IFNα/β signalling pathway [38,39,40], the researchers overexpressed each of the individual WNV NS proteins and the fusion protein NS2B3 in HeLa cells [42]. According to the indirect immunofluorescence (IFA) results, only NS1 could inhibit the NF-κB nuclear transduction; furthermore, the NF-κB nuclear transduction and activation of IRF3 were inhibited in the NS1 stable expression cell lines [42]. In addition, the mRNA and protein levels of cytokine IL6 were inhibited in the NS1 stable expression cell lines [42]. Importantly, the WNV-NS1 protein inhibited the TLR3-induced antiviral effects in plaque experiments [42]. The ability of the TLR3 pathway to inhibit WNV replication remains controversial. Fredericksen and colleagues established a TRIF null model in MEFs to evaluate the ability of the TLR3 pathway to inhibit WNV replication [43]. The results showed that interrupting the TLR3 pathway did not affect WNV replication [43]. The authors also showed that the WNV infection did not interfere with the TLR3 signalling pathway in the TLR3 stable expression cell lines, which was noteworthy. They demonstrated that regardless of whether the cells were treated with poly(I:C) (pIC) during the early or late stages of infection, *IRF3* remained activated. In a 2010 report, the authors speculated that the *NS*1 protein might target TLR3 to abrogate the TLR3 signalling pathway because TLR3 and the *NS*1 protein are both present in the lumen of the ER and endosomes. However, the results of pull-down assays demonstrated no obvious direct interactions between WNV-Uganda (WNV-UG), WNV-New York (WNV-NY), and YFV NS1 and TLR3 [44]. Then, the authors found that YFV NS1 partially colocalized with TLR3, but WNV-UG *NS*1 did not show any colocalization using confocal immunofluorescence microscopy [44]. In a human embryonic kidney (HEK) fibroblast cell line that stably expressed TLR3, the experimental group was transiently transfected with *NS*1 of WNV-UG, WNV-NY, YFV and DENV-2, Thailand and Martinique strains, and relative to the control group, the *IFN*β mRNA levels exhibited a similar amplitude upregulation following stimulation with pIC, while the amount of pIC had only a slight effect on the results [44]. Similarly, *NS*1 from WNV-UG, YFV and DENV2 Thailand did not inhibit the mRNA levels of viperin and ISG56K, which are activated by stimulation with pIC [44]. In general, the *NS*1 proteins derived from WNV, YFV and DENV were transiently transfected into HeLa and HEK293 cells without inhibiting the pIC-mediated *IFN*β and downstream cellular gene production [44]. On the other hand, the authors used pIC to stimulate cell lines that stably expressed WNV-UG *NS*1 and TLR3 and cell lines that stably expressed TLR3, and *NS*1 was also found to be unable to downregulate the TLR3 signalling [44]. The use of different experimental methods can result in differences in the experimental results due to many variable factors. We compared the results in this article with the results reported by Wilson and attributed the differences to three aspects of the experiments, including the different cell lines, the different virus strains, and whether TLR3 is transiently or stably expressed. The different use of cell lines is a potential issue that should be focused on. According to the above experimental analysis, we can conclude that WNV can inhibit TLR signal transduction. Several studies have focused on whether *NS*1 could inhibit TLR signal transduction and whether its targeted molecule was TLR. For example, by investigating whether the NS1 protein inhibits the pIC-mediated activation of IFNβ, Wilson and colleagues transiently transfected NS1 into HeLa cells, performed pIC stimulation for 4 h, and used a reporter system to detect the INFβ transcription levels [42]. Another team used HEK cells to stably express TLR3, transiently transfected them with NS1, stimulated them with pIC for 7.5 h, and performed real-time quantitative RT-PCR for the detection of the IFNβ mRNA levels [44].

### 4.2. Non-Structural Protein 2 (NS2)

NS2 is encoded by the Flaviviridae genome NS2 region. The Flaviviridae NS2 protein can be further processed into the mature proteins NS2A and NS2B (Figure 1). Currently, the function of the NS2 protein remains poorly understood. The HCV NS2 protein is a transmembrane polypeptide; its C-terminus is located in the endoplasmic space, and the N-terminus is located in the cytosol, is cleaved by cell signal peptidase, and is separate from E2/P7 [45]. The specificity of the pestivirus structure, which contains a conserved Cys region, following by a conserved helix, suggests that it interacts with nucleic acids because this structure is primarily found in nucleic acid-binding gene regulatory proteins. Therefore, NS2A may be associated with the regulation of pestivirus genome expression [46]. Researchers have shown that HCV NS2 plays a role in the inhibition of apoptosis [47] and cell growth and interferes with cell cycle regulation, which may be beneficial for viral replication [48].

#### 4.2.1. Suppression of IFN-β Induction

KUNV is a less pathogenic lineage I WNV variant, and its NS2A protein plays an important role in inhibiting the induction of IFNβ promoter-driven transcription [49]. However, NS2A has no known target site for innate immunity.

The DENV non-structural proteins were first found to block the IFN system in 2003 [50]. The pre-treatment and post-treatment of DENV-infected cells with interferon led to the suppression or no suppression of viral replication, respectively, indicating that there is a mechanism employed by the virus to escape the effects of IFN [30]. These strategies are summarized in Table 1. The authors used lentiviral expression plasmids to express the DENV4 non-structural proteins NS2A, NS2B3, NS4A and NS4B to study the mechanism by which these proteins modulate RIG-I/MDA5 /TBK1/IKK/IRF3-mediated signal responses [51]. The results showed that DENV4 NS2A and NS4B dose-dependently inhibited RIG-I/MDA5 /TBK1/IKKε-mediated signalling in HEK293T cells [51]. DENV4 NS2B3 and NS2B3 pro (active, cleaved form) could inhibit STING-mediated signalling, and the co-transfection of the STING and DENV4 non-structural proteins DENV4 NS2B3 and NS2B3 pro could cleave STING according to western blotting assays [51]. However, not all DENV4 non-structural proteins constructed inhibited IRF3-5D (IRF3 phosphomimetic)-mediated signalling, indicating that NS2A and NS4B act upon the signalling pathway at the level of the TBK1/IKKε signalling complexes [51]. These data indicate that DENV4 NS2A and NS4B specifically act on the TBK1/IKKε-directed signalling pathway, but NS2B3 selectively acts on STING [51]. Furthermore, the authors found that NS2A and NS4B could inhibit the phosphorylation of IRF3, and the N-terminal domain (NS4B-Δ-118-260) of DENV4 NS4B was necessary for inhibiting RIG-I/TBK1 signalling [51].

Previous studies have shown that the NS3/4A complex can inhibit RIG-I- and TLR4-mediated signalling, and the authors used NS3/4 as a positive control [15]. Through the use of reporter assays, HCV NS2 was found to inhibit the production of IFN β, IFNα1, IFNλ1, IFNλ3, chemokine CCL5 and CXCL19 [54]. The authors also demonstrated that mutated NS2 protease activity key sites (NS2-H143A and C184A) have no effect on the inhibition of IFNβ. Similarly, the authors found that NS2 could inhibit RIG-I/MAVS/IKKε/TBK1 and TRIF-mediated signalling [54]. Moreover, NS2 can inhibit the phosphorylation of virus-induced IRF3, and following the overexpression of IKKε and TBK1, NS2 can inhibit IRF3 phosphorylation; this effect was dose-dependent, and high-dose expression of NS2 can completely inhibit the TBK1-induced phosphorylation of IRF3 [54]. Importantly, NS2 directly interacted with IKKε and TBK1 according to the GST-Pull down experiment [54].

#### 4.2.2. Suppression of the IFN-Dependent Signalling Pathway

Liu and colleagues demonstrated that KUNV could inhibit the phosphorylation and nuclear transduction of STAT1 and STAT2 [55]. Furthermore, NS2A, NS2B, NS3, NS4A and NS4B could inhibit the phosphorylation and nuclear transduction of STAT2 following the expression of KUNV non-structural proteins [55].

### 4.3. Non-Structural Protein 3 (NS3)

The function of the Flaviviridae NS3 protein has been elucidated. NS3 has been identified as a multifunctional zymoprotein that includes a serine protease [45,56,57,58], a nucleoside triphosphatase (NTPase) [59,60], and an RNA helicase [59,61,62,63,64] which plays an important role in viral replication and interaction with host cells. The sequence of NS3 encodes the serine protease and NTPase/RNA helicase. The former is responsible for the polyprotein cleavage that produces mature viral proteins, which is a prerequisite for virus self-replication and assembly, while the NTPase/RNA helicase is involved in viral RNA replication. DENV NS3 is responsible for the cleavage between NS2A/2B, NS2B3, NS3/4A and NS4B/5, and NS2B is required as a cofactor for its protease activity [65]. The cleavage reaction of HCV NS3 at the three junctions of the non-structural proteins NS3/4A, NS4A/4B and NS4B/NS5A requires the non-structural protein NS4A as a cofactor, and the formation of the complex can ensure a more stable NS3 structure and contribute to the NS3 localization on the membrane [56,61,66,67,68,69,70]. NS3 is involved in the hydrolysis of the capsid protein via its N-terminal 1/3 region; together with NS2B as the protease NS2B3, NS3 completes the hydrolysis, and the NS2B3 and NS3/4A proteases play different roles [71,72].

#### 4.3.1. HCV NS3 Evades Innate Immunity

Many studies have shown that the HCV-NS3 protein is an inhibitor of cellular antiviral responses. Which protein does HCV-NS3 interact with to inhibit the innate immunity signalling pathway? Does this interaction lead to the inhibition of IRF3 phosphorylation? Table 2 summarizes all known underlying mechanisms. Because TLR3 is an important molecule that recognizes double-stranded RNA, it plays an important role in innate immunity. Therefore, the authors studied the inhibition of the TLR3 signalling pathway by NS3, and the reported assays showed that NS3 could inhibit the TRIF- and TBK1-mediated activation of IRF3, which was achieved via an interaction with TBK1 [73]. Furthermore, the authors showed that the binding of the helicase region to TBK1 was necessary by truncating the region expressing the NS3 mutant, including the helicase (nt 541-1893) and protease (nt 1-540) [73]. Furthermore, the authors investigated whether NS3 competes with TBK1 using coIP experiments that inhibited the interaction between TBK1 and IRF3 [73]. The linear ubiquitin chain assembly complex (LUBAC) contains HOIL-1L (heme-oxidized iron regulatory protein ubiquitin ligase-1) and HOIP (HOIL-1L–interacting protein), plays an important role in immune-related studies, and can specifically activate the NF-κB signalling pathway in TNF-α-activated signalling pathways [74]. Because HCV is resistant to TNF-α therapy, HCV-encoded proteins can evade TNF-α mediated signalling. First, the authors demonstrated that HCV can inhibit the TNF-α-mediated activation of NF-κB. After 10 HCV proteins were transiently expressed, the results showed that only NS3 inhibited TNF activation to a large extent. Furthermore, NS3 interacts with LUBAC to inhibit the LUBAC-mediated activation of NF-κB [75].

#### 4.3.2. The Role of Protease Complex *NS*2B3 and *NS*3/4A in Inhibiting Innate Immunity

NS2B3 generally functions as a complex. In the life cycle of DENV, due to host and viral protease processing, its proteins have a complex topology [78]. The Ana Fernandez-Sesma group discovered a novel way by which DENV escapes innate immunization in dendritic cells (DCs), which were the targeted cells along with other cell populations after the host was infected with DENV. The DENV infection induced the activation and release of high levels of proinflammatory cytokines and chemokines, but the activation and release of IFNα/β were minimal. In addition, the cells infected with DENV did not exhibit induced phosphorylation of IRF3, leading to the inhibition of IFNα/β production. Next, their group investigated in-depth the mechanism of DENV-inhibited type I IFN production. The DENV-infected DCs did not produce a strong response to type I interferon inducers, such as NDV (Newcastle disease), SeV (Sendai virus), SFV (Simian foamy virus), or TLR3 ligand poly(I:C). Because NDV is a strong inducer of IFN production and is very sensitive to the anti-viral effect of IFN, the authors established a secondary infection model. The DENV-infected DCs were re-infected with NDV, which did not stimulate the production of IFN; therefore, there is no bystander effect. Therefore, the NS1, NS2A, NS2B3, NS4A and NS4B proteins of DENV were constructed into a Newcastle disease virus (NDV) vector to investigate the non-structural viral proteins in a more realistic environment [79]. Dendritic cells (DCs) were infected with the recombinant NDV vector encoding the DENV proteins, and a slight increase in NDV RNA levels corresponding to NS2B3 and a 35% reduction in the IFN protein levels relative to the control group were observed, suggesting that NS2B3 may increase NDV viral mRNA levels by interfering with the IFN signalling pathway [80]. NS2B3 has also been shown, using reporter assays in 293T-IFNβ-Luc cells, to inhibit Sev- and pIC-induced IFN signalling pathways [80]. S135A and H51A mutations have been shown to affect NS2B3 activity [81,82]. Mutant NS2B3 exhibited a reduced protease activity and reduced inhibition of the IFNβ promoter. Moreover, the final 40 amino acids of NS2B and the first 180 amino acids of NS3 form the NS2B3 helicase centre, which is sufficient to exert a protective effect. Subsequently, the cleavage of several components (i.e., IRF3, IRF7, TLR3, TBK1, IKKε, RIG-I and STING) in the type I IFN signalling pathway were screened to determine the influence of NSB2/3, and the human adaptor molecule STING was identified as a target of the NS2B3 protease complex in human monocyte-derived dendritic cells (MDDCs) [81,82]. The wild-type (WT) NS2B3 cleaves the 42 kDa hSTING protein, but not the mouse version of STING, to a size of approximately 32 kDa, which is consistent with the short region of the hypothetical cleavage site C [52]. This finding may explain the limited replication and spreading of DENV in mice. Furthermore, by performing overexpression experiments in A549 cells, Yu and colleagues demonstrated that DENV NS2B3 can inhibit the activation of IRF3 mediated by JEV, pIC and poly(dA:dT). Furthermore, the cleavage of STING molecules by DENV NS2B3 led to the impaired expression of the IFN and viperin genes. In addition, the presence of residues 93–96 (LRRG) of STING is necessary for DENV NS2B3 to function as a lysogen [53].

HCV NS3/4A plays a more potent role in inhibiting the IFN pathways. HCV NS3/4A could inhibit IRF3 phosphorylation, leading to increased viral replication [77], but the molecular mechanism remains unknown. In 2005, several articles reported the mechanism underlying the role of NS3/4A in innate immunity. Li et al. found that the full-length endogenous MAVS can be cleaved by WT NS3/4A (not mutant NS3/4A), and a truncated MAVS protein containing only the caspase activation and recruitment domain (CARD) and transmembrane (TM) domains was cleaved by the WT NS34A. (E/D) xxxx (C/T) (SA) (x denotes any amino acid) is a conserved sequence for serine protease NS3/4A recognition. MAVS 503EREVP**C**H509 was identified as a target site of NS3/4A [76]. Finally, the authors demonstrated that the endogenous MAVS is cleaved in the replicon cells [76]. The interaction between TLR3 signalling and the NS3/4A component interfered with IRF3 activation by cleaving TRIF at Cys372. Other papers have also demonstrated that NS3/4A inhibited the RIG-I signalling pathway by binding and cleaving MAVS at Cys508 [15].

### 4.4. Non-Structural Proteins 4A and 4B (NS4A and NS4B)

The cleavage of the DENV NS4 protein is initiated by the NS3 protease and host signal peptidase and produces the mature NS4A and NS4B proteins. The cleavage of NS4A/4B is necessary for its inhibition of the IFN signalling pathway [83]. The 2K fragment, which is a linker peptide with 23 amino acids, is located between NS4A/4B in the polypeptide and leads NS4B into the ER membrane. HCV NS4A plays an important role in viral replication and the post-translational processing of polyproteins [66,67,68,69] and can act as a cofactor for the NS3 protease involved in the post-translational processing of the polyprotein. Notably, NS4A is not required for NS3 protease activity and NS5A/5B cleavage, but NS4A significantly increases the cleavage efficiency of the NS3 protease [56].

As previously reported, NS4A inhibited the IFNβ signalling pathway through various mechanisms (Table 3). The authors screened the 10 individual proteins that facilitated the effects of DENV-2 using the ISRE54 reporting system. However, the authors did not identify the specific mechanisms. Expression plasmids for DENV2 NS4A and ISRE-54-CAT were co-transfected into Vero cells for 24 h, and downregulation of the expression of the ISGs was observed in the IFNβ-treated cells, indicating that NS4A interfered with the IFN signalling pathway [50]. In contrast, DENV4 NS4A did not inhibit the transcription of IFNβ, and NS4A from DENV2 did not modulate the IFNβ response as previously described (see Section 4.2.1.). Surprisingly, DENV1 NS4A strongly inhibited the production of RIG-I and TBK1-mediated IFNβ. The author’s conjecture may be related to the six uniquely charged residues of DENV1.

#### 4.4.1. HCV NS4B Inhibits IFN-β Induction by Interacting with STING

The investigations showed that the evasion mechanism of the NS4B protein of HCV involves an interaction with the host cell by targeting STING, but the molecular mechanisms of the NS4B and STING interaction and the blocking of IFN signalling activation are different. Nitta et al. showed, using bimolecular fluorescence complementation (BiFC) assays, that HCV NS4B interacted with STING, showing strong fluorescence compared to that in the corresponding negative controls, thus indicating an obvious protein-protein interaction [84]. As previously reported, STING can bind to MAVS directly to promote the IFN signalling pathway [87]. Therefore, the NS4B protein was expressed with STING or MAVS in HEK293T cells or Huh7 cells, and the binding between STING and MAVS was dose-dependently attenuated by HCV NS4B, as evidenced by immunoprecipitation. Subsequently, by performing a careful comparison, the authors found that the N-terminal domain of several Flaviviridae NS4B shares a structural homology with STING [84,88]. Luciferase assays have shown that the N-terminal 1-84 residues of HCV NS4B are essential for the inhibition of the IFN signalling pathway [61,84]. Ding et al. tested the effects of HCV NS4B on the endogenous STING content and found that even an increased NS4B expression did not affect the level of endogenous STING [85]. Similarly, the formation of STING oligomerization was not affected by NS4B [37,85]. STING oligomerization is important for mediating the interferon signalling pathway [89,90]. Interestingly, the authors demonstrated that the HCV NS4B binding to STING affected the interaction between STING and TBK1 in PH5CH8 cells rather than the interaction between STING and MAVS [85]. This finding is inconsistent with the previous study. The authors attributed this finding to the dynamic changes in the mitochondria-associated ER membrane (MAM) structure. The MAM is a dynamic structure in which the mitochondria and the MAVS-STING association mainly reside [91,92]. Yi et al. showed that HCV NS4B dose-dependently suppressed the STING-mediated IFN signalling pathway using luciferase assays and a coIP experiment in 293T and Huh7.5 cells. Similar results showed that NS4B dose-dependently inhibited the endogenous STING content, and four predicted transmembrane regions of HCV NS4B were mainly responsible for the suppression of STING accumulation [86].

#### 4.4.2. NS4B Inhibits the IFN-Dependent Signalling Pathway

Munoz-Jordan and colleagues reported that DENV2 NS4B inhibited IFN-mediated STAT1 phosphorylation [50]. The authors also investigated which domain of DENV2 NS4B is essential for the inhibition of the IFN signalling pathway. The authors found that the first 125 amino acids of DENV NS4B are sufficient for the inhibition of α/β IFN signalling, and NS4B did not inhibit the IFN-stimulated activation of the ISRE promoter in the absence of the 2K fragment; however, the subcellular localization of NS4B without 2K did not change [83]. The abovementioned evidence suggested that the absence of the 2K fragment led to an abnormal folding and topological changes in NS4B, rather than affecting the NS4B ER localization [83]. The function of NS4B in suppressing the IFN pathway is conserved. NS4B from DENV, YFV and WNV with STAT1 showed colocalization in the cytoplasm, and NS4B could inhibit the activation of ISRE mediated by IFNβ [83].

### 4.5. Non-Structural Protein 5 (NS5)

NS5 is encoded by the C terminus of the Flaviviridae genome. The protein cleavage of HCV NS5 is initiated by the NS3 protein and produces the mature proteins NS5A and NS5B. NS5B (or NS5) is an RdRp (RNA-dependent RNA polymerases) that is involved in viral replication [93,94,95,96]. In addition to the RdRp activity, the HCV *NS5B* gene has preferential TNTase (terminal nucleotide transferase) activity with Uridine triphosphate (UTP) as a substrate [95,97]. The 5′ end of the genome of the flavivirus has a type I CAP structure, which is closely related to the MTase (methyltransferase) activity of the NS5 protein. The functional region of MTase is located at the amino terminus of NS5, with a conservative sequence motif K61-D146-K182-E218, in which 2′-O methylation requires a complete sequence, while N-7 methylation requires only D146, and the other three amino acids play a synergistic effect [98,99].The C terminus of the Flaviviridae polyprotein (NS5 or the C terminal of NS5B) encodes a polyprotein that has a GDD sequence (Gly-Asp-Asp) that is conserved among all currently identified RdRps [100,101]. The HCV NS5A protein has a nuclear localization sequence, that is, PPRKKRTVV, but the subcellular localization of the NS5A protein in live samples from patients with chronic HCV infection indicates that the NS5A protein is located in the cytoplasm; the nuclear localization signal sequence is functional and can lead other heterologous proteins, such as E. coli β-galactosidase, into the nucleus [102]. The exact function of NS5A in viral replication is not well understood and remains to be explored.

#### 4.5.1. HCV NS5A Inhibits IFNβ Induction

The NS5 proteins of Flaviviridae can inhibit IFN signalling, including DENV, JEV, TBEV, YFV, ZIKV and HCV (Table 4) [103,104,105,106,107,108,109,110,111,112,113,114,115,116,117]. HCV NS5A impaired TLR-MyD88 signalling by binding to MyD88. Amino acid residues 240 to 280 of NS5A, formerly identified as the interferon sensitivity-determining region (ISDR), interacted with the death domain of MyD88 and inhibited the recruitment of interleukin-1 receptor-associated kinase 1 (IRAK1) to MyD88 [108]. Amit Raychoudhuri and colleagues suggested that the HCV NS5A protein impaired the nuclear translocation of IRF7 after MyD88 activation, but the underlying mechanism remains unknown [109]. Recently, the authors observed that the HCV NS5A protein interacted with IRF7 and blocked IFNα promoter activity. The amino acid residues Arg216 and Arg217 of NS5A played an important role in this function [104].

#### 4.5.2. Flaviviridae NS5 Inhibits the IFN-Dependent Signalling Pathway

In the IFN-mediated signalling pathway, the Flaviviridae NS5 protein antagonized IFN signalling by binding and degrading STAT1 or STAT2 (Figure 2). Using the ISRE and γ interferon activation site (GAS) reporting systems, the authors found that LGTV inhibited the IFNα- and IFNγ-mediated JAK-STAT pathways due to the inhibition of STAT1 and STAT2 [105]. After LGTV non-structural proteins were expressed, the results showed that only NS5 could inhibit JAK-STAT signalling, and NS5 could inhibit STAT1 phosphorylation by western blot through coIP, NS5 was shown to interact with the IFN receptor [105]. The HCV NS5A C terminus (aa residues 237 to 447) physically interacted with STAT1, which led to a decrease in phosphorylated STAT 1 (p-STAT1) and the inhibition of the IFNα signalling pathway [87]. In addition to STAT1, the DENV NS5 protein could degrade STAT2 as a polyprotein undergoing proteolytic processing for its maturation [103,116]. Because YFV and DENV belong to the flavivirus family, both are transmitted through mosquitoes and can cause fever. Laurent-Rolle and colleagues determined whether YFV and DENV have similar mechanisms for inhibiting the IFN signalling pathway. Interestingly, YFV and DENV can inhibit the IFN signalling pathway, but YFV-NS5 binds to STAT2 only when the cells are stimulated with type I interferon (IFN-I) [106]. The expression of the ZIKV NS5 alone resulted in the proteasomal degradation of STAT2, which was species-specific to humans but not mice, which may explain the requirement of IFN deficiency to observe Zika-induced disease in mice [107]. ZIKV can block the response of type I IFN by inhibiting the phosphorylation of STAT1 and STAT2 when infecting human DC cells. Interestingly, ZIKV virus can promote the IFN transcription level increasing, but the content of IFN protein is very low, and ZIKV can greatly induce the expression level of antiviral effectors. The relationship between ZIKV, type I interferon signalling and antiviral proteins require more research to accurately define. The HCV NS5A protein also directly interacts with key antiviral molecules, such as the PKR protein kinase and 2′-5′-oligoadenylate synthetase (2′-5′ OAS), leading to the inhibition of IFN antiviral activity, which may be the mechanism by which HCV escapes the antiviral effects of IFN [110,111]. WNV NS5 protein MTase plays an important role in immune escape, and this mode of action provides a new way for our research. 2′-O methylation of the 5′ cap of viral RNA has little effect on viral replication, but the virulence of the virus has decreased significantly after mutations in its key site (E218A). Further studies have shown that NS5 protein 2′-O methylation can down-regulate the expression of IFIT [117,118]. In contrast to other Flaviviridae proteins, JEV-NS5 activated the protein tyrosine phosphatase (PTP), which is identified as a negative regulator of the JAK-STAT signalling pathway, resulting in the inhibition of STAT1 and TYK2 phosphorylation and STAT1 nuclear translocation [115]. Through a truncation analysis, the 83 amino acids at the N-terminus of JEV-NS5 were determined to be indispensable for its inhibition of IFN signalling [115].

## 5. Conclusions

After long-term co-evolution of the virus and host, Flaviviridae has developed many strategies to evade the host’s innate immunity. First, the virus inhibits the induction of IFN. WNV NS1 and KUNV NS2A act on the TLR3 and RIG-I pathways, respectively, and DENV NS2B3 cleavage of STING results in the suppression of IFNβ. Then, the virus inhibits the expression of antiviral molecules. DENV NS4B and TBEV NS5 inhibit the IFN-mediated phosphorylation of STAT. Finally, the virus abolishes the functional activity of antiviral molecules. The interaction between HCV NS5 and PKR or 2′-5′ OAS inhibits the antiviral function of the ISGs. The Flaviviridae non-structural proteins play an important role in immune escape, but the signalling pathway and target sites of many non-structural or structural proteins remains unknown. Importantly, it is not known how they work together to interfere with the innate immune system. Future studies should not only deepen the understanding of immune evasion by Flaviviridae but also provide target sites for the development of new vaccines.

## Figures and Tables

**Table 1:** Immune evasion by flaviviridae NS2.

Virus	Protein	Immune Evasion Mechanisms	Reference
DENV	NS2A	Inhibit RIG-I/MAVS signaling by blocking TBK1/IRF3 phosphorylation	[51]
DENV	NS2B3	Inhibit type I IFN production by cleaving human STING	[52]
		Inhibit innate immunity by cleaving STING	[53]
HCV	NS2	Interact with IKKε and TBK1 leading the inhibition of IRF3 phosphorylation	[54]
KUN	NS2A	Inhibit the induction of IFN-β promoter-driven transcription	[49]

**Table 2:** Immune evasion by flaviviridae NS3.

Virus	Protein	Immune Evasion Mechanisms	Reference
HCV	NS3	Interact with TBK1 leading the IRF3 inhibition	[73]
		Interact with LUBAC leading the inhibition of NF-κB activation	[75]
HCV	NS3/4A	Inhibit TLR3 signaling pathway by cleaving TRIF	[76]
		Block the IRF3 phosphorylation	[77]
		Inhibit RLR signaling by binding and cleaving MAVS	[15,76]

**Table 3:** Immune evasion by flaviviridae NS4.

Virus	Protein	Immune Evasion Mechanisms	Reference
DENV	NS4A	Inhibit IFNβ mediated ISRE54 promoter activation	[50]
HCV	NS4B	Compete with STING for binding to MAVS to influence RLR signaling	[84]
		Compete with STING for binding to TBK1 to influence RLR signaling	[85]
		Inhibit STING accumulation led the suppression of RLR signaling	[86]
WNV/DENV	NS4B	Inhibit RIG-I/MAVS signaling by blocking TBK1/IRF3 phosphorylation	[51]
DENV	NS4B	Inhibit IFN-mediated STAT1 phosphorylation	[50]
YFV/WNV/DENV	NS4B	Inhibit the activation of ISRE by IFNβ stimulation	[51,83,87]

**Table 4:** Immune evasion by flaviviridae *NS*5.

Virus	Protein	Immune Evassion Mechanisms	Reference
HCV	NS5A	Impair TLR-MyD88 signaling via binding to MyD88	[19]
		Impair the nuclear translocation of IRF7 after MyD88 activation	[104]
		Bind STAT1 to inhibit JAK/STAT signaling pathway	[87]
		Bind to PKR and inhibit its activity	[110]
		Inhibit 2′,5′-oligoadenylate synthetase function	[111]
WNV	NS5	Impair IFN-mediated JAK-STAT signaling by suppressing STAT1 phosphorylation	[112]
DENV	NS5	Inhibit IFN-mediated signaling by blocking STAT2 phosphorylation	[113]
YFV	NS5	Inhibit type I IFN mediated signaling by binding to STAT2	[106]
ZIKV	NS5	Target STAT2 to inhibit Type I interferon signaling	[107]
LGTV	NS5	Inhibit the JAK-STAT signaling by interacting with IFNAR2/IFNAR1	[105]
TBEV	NS5	Impair JAK/STAT signaling by blocking STAT1 phosphorylation	[114]
JEV	NS5	Impair JAK-STAT signaling by blocking STAT1 and Tyk2 phosphorylation	[115]
